# Long-term treatment with the pan-PPAR agonist tetradecylthioacetic acid or fish oil is associated with increased cardiac content of n-3 fatty acids in rat

**DOI:** 10.1186/1476-511X-11-82

**Published:** 2012-06-27

**Authors:** Elin Strand, Bodil Bjorndal, Ottar Nygard, Lena Burri, Christ Berge, Pavol Bohov, Bjørn J Christensen, Kjetil Berge, Hege Wergedahl, Asgaut Viste, Rolf K Berge

**Affiliations:** 1 Institute of Medicine, University of Bergen, Haukeland University Hospital, Laboratory Building 8th floor, 5021, Bergen, Norway; 2 Department of Heart Disease, Haukeland University Hospital, 5021, Bergen, Norway; 3 Department of Surgery, Haukeland University Hospital, 5021, Bergen, Norway; 4 Faculty of Education, Section for food and health, Bergen University College, 5096, Bergen, Norway; 5 Department of Surgical Sciences, University of Bergen, 5021, Bergen, Norway

**Keywords:** Heart metabolism, Peroxisome proliferator-activated receptor, Polyunsaturated fatty acids, Tetradecylthioacetic acid

## Abstract

**Background:**

Excess peroxisome proliferator-activated receptor (PPAR) stimulation has been associated with detrimental health effects including impaired myocardial function. Recently, supplementation with n-3 polyunsaturated fatty acids (PUFA) has been associated with improved left ventricular function and functional capacity in patients with dilated cardiomyopathy. We investigated the long-term effects of the pan-PPAR agonist tetradecylthioacetic acid (TTA) and/or high-dose fish oil (FO) on cardiac fatty acid (FA) composition and lipid metabolism. Male Wistar rats were given one out of four different 25% (w/v) fat diets: control diet; TTA diet; FO diet; or diet containing both TTA and FO.

**Results:**

After 50 weeks n-3 PUFA levels were increased by TTA and FO in the heart, whereas liver levels were reduced following TTA administration. TTA was associated with a decrease in arachidonic acid, increased activities of carnitine palmitoyltransferase II, fatty acyl-CoA oxidase, glycerol-3-phosphate acyltransferase, and fatty acid synthase in the heart. Furthermore, cardiac *Ucp3* and *Cact* mRNA was upregulated.

**Conclusions:**

Long-term treatment with the pan-PPAR agonist TTA or high-dose FO induced marked changes in PUFA composition and enzymatic activity involved in FA metabolism in the heart, different from liver. Changes included increased FA oxidation and a selective increase in cardiac n-3 PUFA.

## Background

Peroxisome proliferator-activated receptors (PPAR) are members of the nuclear hormone receptor family consisting of three subtypes (α, δ, and γ) with distinct and overlapping expression patterns [1]. PPARα seems to be the primary transcription regulator of enzymes involved in fatty acid (FA) oxidation in the heart. A substrate switch from FA to glucose is thought to be a result of decreased PPARα activity [2]. The healthy heart generates most of its energy as ATP through FA catabolism, which primarily occurs in mitochondria, but also to a small extent in peroxisomes [3]. Thus, a continuous FA supply to the heart is important to sustain the contractile activity as this tissue can store and synthesize FA only to a limited extent [4]. Excess PPARα stimulation may, however, have detrimental effects [5] including substrate overload of FA in the tissue, which is also associated with conditions like obesity and insulin resistance [6]. Myocardial dysfunction could hereby be the result of an obesity-associated reduced glucose and increased FA utilization in heart [7,8]. Lipotoxicity affects mitochondrial function through altered protein phosphorylation and increased levels of cytotoxic intermediate products of β-oxidation, which could lead to mitophagy (controlled degradation of mitochondria) or apoptosis [9].

A diet rich in n-3 polyunsaturated FA (PUFA) like eicosapentaenoic acid (EPA, C20:5n-3) and docosahexaenoic acid (DHA, C22:6n-3), has been associated with reduced triacylglycerol (TAG) levels, anti-inflammatory effects, and a lowered risk of cardiovascular disease and mortality [10,11]. These beneficial effects may be less evident in patients with stable angina [12,13], but particularly pronounced in patients with reduced ventricular function and heart failure [14]. Very high doses of n-3 PUFA could, however, be pro-inflammatory and pro-oxidative [15].

Fibrates are a group of specific PPARα-targeted drugs being utilized during the past three decades due to their TAG-reducing effects. Their clinical gain, however, still remains to be demonstrated [16,17], and their routine use in combined hyperlipidemia has recently been abandoned [18]. Thus, excess PPARα activation may be associated with unfavorable metabolic effects that could counteract the apparent beneficial effects on lipid metabolism and inflammation.

Similarly to PUFA and fibrates, tetradecylthioacetic acid (TTA) has an especially pronounced affinity to PPARα [19]. TTA is a saturated FA analogue with 16 carbon atoms and one sulphur atom at position three, belonging to a group of sulphur-substituted FA (3-thia FA) with pan-PPAR activation properties [20]. It is known to reduce plasma TAG, probably due to hepatic proliferation of mitochondria and an increased β-oxidation of FA through PPAR-dependent mechanisms [21].

To this date inconsistent results from only a limited number of previous studies on the effects of PPARα agonists on cardiac substrate metabolism exist [22-27]. We hypothesized that treatment with the pan-PPAR agonist TTA and/or high-dose FO, would influence cardiac lipid metabolism and thereby affect FA composition. Data from liver was also applied to reveal organ specific changes in the heart.

## Results

### Animal body weight and cardiac lipids

Mean (±SD) weight of the animals at study start was 266 (±32) g, and there were no significant weight differences between groups. During the study animals receiving TTA or the combination of TTA and FO had significantly reduced body weight gains compared to control (p < 0.001 for both groups). In addition, all groups had an average comparable feed intake [28]. To eliminate the possibility of an effect-modification caused by the jejuno-gastric reflux surgical procedure, changes in body weight and plasma lipids were studied in an 11 week pilot study. This study did not reveal any statistically significant differences in neither body weight nor plasma lipids when comparing operated animals to controls.

After 50 weeks of dietary intervention there was no change in cardiac TAG as a result of TTA or FO treatment. There was, however, a significant increase in total cholesterol after TTA (mean ± SD, 3.21 ± 0.20 vs. 2.93 ± 0.24 μmol/g heart tissue, p < 0.001), and in phospholipids after FO (14.68 ± 0.91 vs. 13.69 ± 0.88 μmol/g heart tissue, p < 0.001) treatment.

### Changes in cardiac fatty acids following TTA and/or FO administration

**Figure 1:**
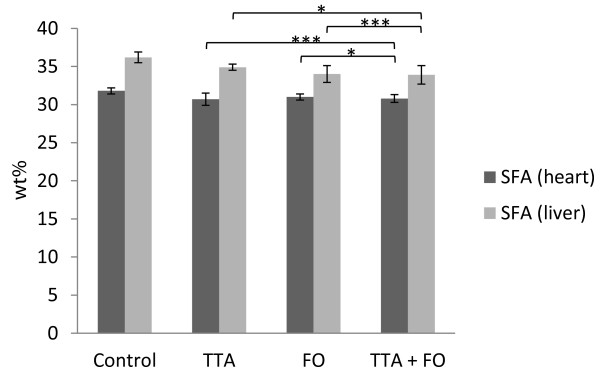
**Saturated fatty acids (SFA) (wt%) in heart and liver.** Levels of SFA in heart (dark grey bars) and liver (light grey bars) and distribution in diet groups were as indicated. Values are mean ± SD. Asterix indicates statistical significance of variance ratio and effects of TTA (long clamps) and FO (short clamps) (*p < 0.05; ***p < 0.001).

Two-way ANOVA indicated a significant increase in total FA (p = 0.002) in the heart of animals receiving TTA (Table 1). Total saturated FA (SFA) were decreased following TTA treatment (p < 0.001; Figure 1), while monounsaturated FA (MUFA) were decreased following FO treatment (p = 0.001; Table 1). The interaction (TTA*FO) for SFA was borderline significant in heart (p = 0.01). The level of mead acid (MA, C20:3n-9) was elevated following TTA treatment (p < 0.001) and decreased in animals receiving FO (p < 0.001; Table 1).

**Table 1:** Fatty acid composition (wt%) in heart of rats after 50 weeks of diet administration

		**Dietary supplementation**^**a**^			**Statistical significance of variance ratio (P)**^**b**^**, effects of**		
	**Control**	**TTA**	**FO**	**TTA + FO**	**TTA**	**FO**	TTA*FO
Total FA (μg/g tissue)	22196 ± 2706^c^	24087 ± 1394	22578 ± 1503	24185 ± 1613	0.002	0.66	0.79
C16:0	11.1 ± 1.3	11.0 ± 1.1	10.0 ± 1.2	10.8 ± 0.6	0.25	0.05	0.22
C18:0	19.7 ± 1.2	18.8 ± 1.1	19.8 ± 1.1	18.9 ± 0.9	0.005	0.55	1.00
MUFA	10.2 ± 3.6	11.2 ± 3.1	8.4 ± 3.0	6.7 ± 2.6	0.70	0.001	0.14
C16:1n-7	0.24 ± 0.11	0.32 ± 0.11	0.21 ± 0.10	0.16 ± 0.09	0.70	0.002	0.03
C18:1n-7	2.6 ± 0.1	1.8 ± 0.2	2.4 ± 0.2	2.1 ± 0.2	<0.001	0.79	<0.001
C16:1n-9	0.10 ± 0.05	0.15 ± 0.04	0.11 ± 0.06	0.10 ± 0.04	0.27	0.13	0.09
C18:1n-9 (OA)	6.9 ± 3.4	8.5 ± 2.9	5.2 ± 2.7	3.9 ± 2.3	0.85	<0.001	0.08
C20:3n-9 (MA)	0.10 ± 0.01	0.55 ± 0.28	0.06 ± 0.01	0.05 ± 0.01	<0.001	<0.001	<0.001
C18:2n-6 (LA)	15.7 ± 1.0	17.2 ± 2.0	9.2 ± 1.0	5.8 ± 1.2	0.03	<0.001	<0.001
C18:3n-3 (ALA)	0.15 ± 0.06	0.20 ± 0.06	0.14 ± 0.06	0.10 ± 0.06	0.57	0.004	0.02
C18:4n-3	0.4 ± 0.3^k^	0.3 ± 0.3^k^	82.8 ± 38.0^k^	89.4 ± 30.4^k^	0.64	<0.001	0.64
TTA	ND	2.8 ± 1.0	ND	1.7 ± 0.5			
TTA:1n-8	ND	0.79 ± 0.49	ND	0.12 ± 0.04			
n-3 PUFA/n-6 PUFA ratio	0.39 ± 0.04	0.55 ± 0.13	1.6 ± 0.2	3.9 ± 1.1	<0.001	<0.001	<0.001
Δ5 desaturase index (n-6)^d^	49.0 ± 4.6	9.8 ± 1.8	38.5 ± 5.8	10.6 ± 1.5	<0.001	<0.001	<0.001
Δ5 desaturase index (n-3)^e^	2.6 ± 0.4	4.2 ± 0.7	24.5 ± 4.3	36.4 ± 7.0	<0.001	<0.001	<0.001
Δ6 desaturase index (n-6)^f^	1.1 ± 0.4^k^	2.6 ± 0.7^k^	2.5 ± 0.7^k^	7.9 ± 2.0^k^	<0.001	<0.001	<0.001
Δ6 desaturase index (n-3)^g^	2.7 ± 2.3^k^	1.7 ± 1.4^k^	0.61 ± 0.10	0.94 ± 0.19	<0.001	<0.001	<0.001
Δ9 desaturase index (C16)^h^	0.02 ± 0.01	0.03 ± 0.01	0.02 ± 0.01	0.01 ± 0.01	0.73	0.001	0.003
Δ9 desaturase index (C18)^i^	0.36 ± 0.22	0.46 ± 0.18	0.27 ± 0.16	0.21 ± 0.14	0.71	0.001	0.13
Anti-inflammatory index^j^	68.8 ± 6.7	155 ± 37.6	274 ± 50.1	866 ± 234	<0.001	<0.001	<0.001

Abbreviations: TTA, tetradecylthioacetic acid; FO, fish oil; FA, fatty acids; MUFA, monounsaturated fatty acids; OA, oleic acid; MA, mead acid; LA, linoleic acid; ALA, α-linolenic acid; PUFA, polyunsaturated fatty acids; ND, not detectable.

^a^ n=12 in each group.

^b^ P-values from two-way ANOVA.

^c^ Values are mean ± SD.

^d^ C20:4n-6/C20:3n-6 (an indirect index of Δ5 desaturase activity based on n-6 PUFA).

^e^ C20:5n-3/C20:4n-3 (an indirect index of Δ5 desaturase activity based on n-3 PUFA).

^f^ C18:3n-6/C18:2n-6 (an indirect index of Δ6 desaturase activity based on n-6 PUFA).

^g^ C18:4n-3/C18:3n-3 (an indirect index of Δ6 desaturase activity based on n-3 PUFA).

^h^ C16:1n-7/C16:0 (an indirect index of Δ9 desaturase activity based on C16 SFA/MUFA).

^i^ C18:1n-9/C18:0 (an indirect index of Δ9 desaturase activity based on C18 SFA/MUFA).

^j^ ((C22:6n-3 + C22:5n-3 + C20:3n-6 + C20:5n-3)/C20:4n-6)*100.

^k^ Values are *10^-3^.

**Figure 2:**
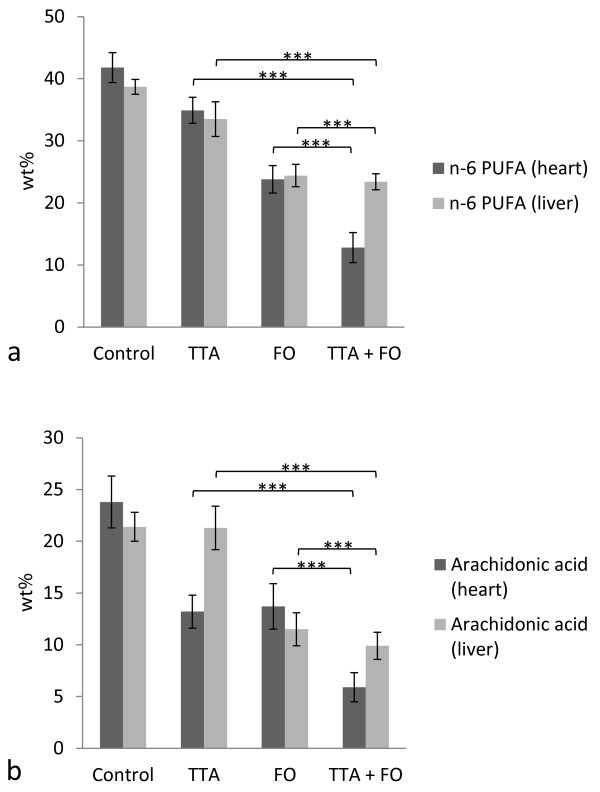
**n-6 polyunsaturated fatty acids (PUFA) (wt%) in heart and liver.** Levels of n-6 PUFA (**a**) and arachidonic acid (**b**) in heart (dark grey bars) and liver (light grey bars) and distribution in diet groups were as indicated. Values are mean ± SD. Asterix indicates statistical significance of variance ratio and effects of TTA (long clamps) and FO (short clamps) (***p < 0.001).

Total cardiac n-6 PUFA were decreased after both TTA and FO treatment (p < 0.001 for both; Figure 2a). The decrease in total n-6 PUFA after TTA treatment seemed to be due to a decrease in arachidonic acid (ARA) levels (p < 0.001; Figure 2b), while most other measured n-6 PUFA were increased (Additional file 1). The interaction (TTA*FO) was significant for total n-6 PUFA (p = 0.003) and borderline significant for ARA (p = 0.02).

**Figure 3:**
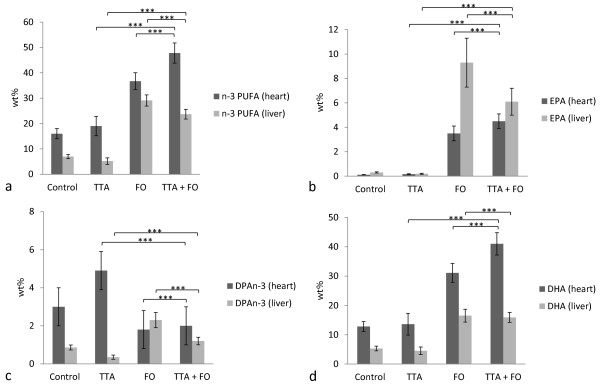
**n-3 polyunsaturated fatty acids (PUFA) (wt%) in heart and liver.** Levels of n-3 PUFA (**a**), eicosapentaenoic acid (EPA) (**b**), docosapentaenoic acid n-3 (DPAn-3) (**c**), and docosahexaenoic acid (DHA) (**d**) in heart (dark grey bars) and liver (light grey bars) and distribution in diet groups were as indicated. Values are mean ± SD. Asterix indicates statistical significance of variance ratio and effects of TTA (long clamps) and FO (short clamps) (***p < 0.001).

There was a concomitant enrichment of total n-3 PUFA after TTA and FO treatment (p < 0.001 for both; Figure 3a). The total increase in n-3 PUFA after TTA administration was caused by significantly increased levels of EPA (Figure 3b), docosapentaenoic acid (n-3) (DPAn-3) (Figure 3c), and DHA (Figure 3d) (p < 0.001 for all), where DPAn-3 accounted for the foremost increase. After FO treatment, on the other hand, DPAn-3 levels decreased (p < 0.001), while EPA and DHA increased (<0.001 for both). The interaction (TTA*FO) was significant for total n-3 PUFA, EPA, DPAn-3, and DHA in heart (p < 0.001 for all). Total PUFA composition is provided as a supplementary table (Additional file 1).

As expected, the n-3/n-6 PUFA ratio was greatly increased following FO supplementation (p < 0.001). However, this ratio was also significantly increased after TTA treatment (p < 0.001; Table 1).

Desaturase activities were calculated as ratios of FA, which represent indirect activity indexes. TTA treatment led to a marked decrease in n-6 (p < 0.001) and an increase in n-3 (p < 0.001) Δ5 desaturase indexes. There was a concomitant increase in n-6 (p < 0.001) and decrease in n-3 (p < 0.001) Δ6 desaturase indexes. Furthermore, the anti-inflammatory index was increased for both supplements (p < 0.001; Table 1).

### Changes in liver fatty acids following TTA and/or FO administration

As the liver is of crucial importance for FA metabolism, liver composition was also included for comparison (Table 2). There was a significant increase in C16:0 and a decrease in C18:0 levels in liver after dietary intervention with TTA (p < 0.001 for both), while FO decreased total SFA (p < 0.001). Total MUFA were increased after TTA and decreased following FO treatment (p < 0.001 for both). As in heart muscle, liver MA was elevated following TTA treatment and decreased in animals receiving FO (p < 0.001 for both).

**Table 2:** Fatty acid composition (wt%) in liver of rats after 50 weeks of diet administration

		**Dietary supplementation**^**a**^			**Statistical significance of variance ratio (P)**^**b**^**, effects of**		
	**Control**	**TTA**	**FO**	**TTA + FO**	**TTA**	**FO**	TTA*FO
Total FA (μg/g tissue)	42287 ± 4330^c^	39177 ± 2734	43285 ± 4935	41488 ± 4163	0.10	0.27	0.66
C16:0	18.8 ± 0.7	19.8 ± 1.2	16.9 ± 0.6	19.5 ± 0.7	<0.001	0.001	0.01
C18:0	16.1 ± 1.2	14.1 ± 1.0	16.0 ± 0.8	13.3 ± 1.2	<0.001	0.22	0.38
MUFA	17.8 ± 2.2	22.4 ± 3.1	12.4 ± 1.5	17.0 ± 1.6	<0.001	<0.001	0.99
C16:1n-7	0.61 ± 0.38	0.69 ± 0.30	0.37 ± 0.11	0.40 ± 0.08	0.52	0.007	0.76
C18:1n-7	1.7 ± 0.3	1.4 ± 0.1	1.2 ± 0.1	1.1 ± 0.0	<0.001	<0.001	0.09
C16:1n-9	0.20 ± 0.06	0.41 ± 0.07	0.20 ± 0.03	0.37 ± 0.08	<0.001	0.45	0.33
C18:1n-9 (OA)	14.7 ± 1.8	19.5 ± 2.9	10.0 ± 1.4	14.7 ± 1.6	<0.001	<0.001	0.95
C20:3n-9 (MA)	0.23 ± 0.02	1.15 ± 0.45	0.10 ± 0.01	0.15 ± 0.03	<0.001	<0.001	<0.001
C18:2n-6 (LA)	15.5 ± 1.3	9.3 ± 2.0	11.9 ± 0.6	11.7 ± 0.7	<0.001	0.19	<0.001
C18:3n-3 (ALA)	0.35 ± 0.06	0.08 ± 0.03	0.34 ± 0.06	0.17 ± 0.03	<0.001	0.03	0.006
C18:4n-3	0.02 ± 0.00	0.01 ± 0.00	0.12 ± 0.03	0.07 ± 0.01	<0.001	<0.001	0.002
TTA	ND	1.8 ± 0.4	ND	1.3 ± 1.1			
TTA:1n-8	ND	0.97 ± 0.18	ND	0.56 ± 0.17			
n-3 PUFA/n-6 PUFA ratio	0.18 ± 0.02	0.16 ± 0.04	1.20 ± 0.18	1.02 ± 0.13	0.02	<0.001	0.05
Δ5 desaturase index (n-6)^d^	29.6 ± 8.1	11.8 ± 2.1	24.5 ± 5.2	7.8 ± 1.1	<0.001	0.02	0.77
Δ5 desaturase index (n-3)^e^	3.6 ± 0.6	3.4 ± 0.9	34.9 ± 5.1	35.5 ± 6.3	0.91	<0.001	0.78
Δ6 desaturase index (n-6)^f^	14.0 ± 3.2^k^	47.9 ± 14.9^k^	4.4 ± 1.2^k^	16.9 ± 2.8^k^	<0.001	<0.001	0.001
Δ6 desaturase index (n-3)^g^	18.1 ± 3.9	9.8 ± 3.5	3.0 ± 0.5	2.5 ± 0.3	<0.001	<0.001	<0.001
Δ9 desaturase index (C16)^h^	0.03 ± 0.02	0.03 ± 0.01	0.02 ± 0.01	0.02 ± 0.00	0.92	0.01	0.66
Δ9 desaturase index (C18)^i^	0.92 ± 0.17	1.40 ± 0.29	0.63 ± 0.11	1.12 ± 0.24	<0.001	0.001	0.90
Anti-inflammatory index^j^	34.0 ± 2.8	32.7 ± 7.5	254 ± 53.5	253 ± 47.1	0.91	<0.001	0.99

Abbreviations: TTA, tetradecylthioacetic acid; FO, fish oil; FA, fatty acids; MUFA, monounsaturated fatty acids; OA, oleic acid; MA, mead acid; LA, linoleic acid; ALA, α-linolenic acid; PUFA, polyunsaturated fatty acids; ND, not detectable.

^a^ n=8 in each group.

^b^ P-values from two-way ANOVA.

^c^ Values are mean ± SD.

^d^ C20:4n-6/C20:3n-6 (an indirect index of Δ5 desaturase activity based on n-6 PUFA).

^e^ C20:5n-3/C20:4n-3 (an indirect index of Δ5 desaturase activity based on n-3 PUFA).

^f^ C18:3n-6/C18:2n-6 (an indirect index of Δ6 desaturase activity based on n-6 PUFA).

^g^ C18:4n-3/C18:3n-3 (an indirect index of Δ6 desaturase activity based on n-3 PUFA).

^h^ C16:1n-7/C16:0 (an indirect index of Δ9 desaturase activity based on C16 SFA/MUFA).

^i^ C18:1n-9/C18:0 (an indirect index of Δ9 desaturase activity based on C18 SFA/MUFA).

^j^ ((C22:6n-3 + C22:5n-3 + C20:3n-6 + C20:5n-3)/C20:4n-6)*100.

^k^ Values are *10^-3^.

Total liver n-6 PUFA were decreased after TTA and FO administration (p < 0.001, Figure 2a). Furthermore, n-3 PUFA were increased after FO (p < 0.001) but decreased after TTA (p < 0.001) treatment (Figure 3a). This decrease in n-3 PUFA in liver after TTA treatment was the opposite of what was seen in heart muscle (Figure 3a). The interaction (TTA*FO) was significant for n-6 PUFA (p = 0.004), n-3 PUFA (p = 0.004), EPA (p < 0.001), and DPAn-3 (p = 0.001).

Main changes in estimated desaturase indexes in liver were similar to changes in heart, except from an increase in Δ9 desaturase activity (C18 ratio) after TTA (p < 0.001) administration (Table 2). The hepatic anti-inflammatory index remained unchanged after TTA treatment.

### Changes in the activities of key metabolic enzymes

TTA treatment was associated with reduced activity of carnitine palmitoyltransferase (CPT)-I in the heart (p < 0.001; Table 3), but the effect was less pronounced in the presence of malonyl-CoA (the natural inhibitor of CPT-I) (p < 0.001). The activity of CPT-II was increased after TTA treatment (p < 0.001; Table 3). FO treatment did not affect the activities of CPT-I or CPT-II.

**Table 3:** Enzyme activity (nmol/min/mg protein) in heart of rats after 50 weeks of diet administration

		**Dietary supplementation**^**a**^			**Statistical significance of variance ratio (P)**^**b**^**, effects of**		
	**Control**	**TTA**	**FO**	**TTA + FO**	**TTA**	**FO**	TTA*FO
CPT-I	2.79 ± 0.26^c^	1.69 ± 0.38	2.54 ± 0.12	1.75 ± 0.14	<0.001	0.22	0.06
CPT-I (with malonyl-CoA)	1.89 ± 0.21	1.23 ± 0.28	1.77 ± 0.14	1.31 ± 0.13	<0.001	0.73	0.14
CPT-II	10.79 ± 1.03	23.55 ± 3.15	12.40 ± 2.33	22.39 ± 4.32	<0.001	0.82	0.15
ACOX	1.65 ± 0.31	4.53 ± 1.16	4.61 ± 1.41	8.47 ± 1.37	<0.001	<0.001	0.19
GPAT	0.25 ± 0.06	0.43 ± 0.09	0.39 ± 0.09	0.64 ± 0.14	<0.001	<0.001	0.37
FAS	0.04 ± 0.01	0.09 ± 0.01	0.06 ± 0.02	0.10 ± 0.02	<0.001	0.01	0.34

Abbreviations: TTA, tetradecylthioacetic acid; FO, fish oil; CPT-I, carnitine palmitoyltransferase I; CPT-II, carnitine palmitoyltransferase II; ACOX, fatty acyl-CoA oxidase; GPAT, glycerol-3-phosphate acyltransferase; FAS, fatty acid synthase.

^a^ n=10 in each group.

^b^ P-values from two-way ANOVA.

^c^ Values are mean ± SD.

Enzyme activities of fatty acyl-CoA oxidase (ACOX) and glycerol-3-phosphate acyltransferase (GPAT) in heart tissue were induced by TTA and FO (p < 0.001 for all), while fatty acid synthase (FAS) was induced by TTA (p < 0.001).

### PPAR-targeted genes and cardiac expression at the mRNA level

A total of 16 different PPAR-targeted genes were selected and gene expression was measured with real-time PCR (qPCR) on total RNA isolated from heart tissue. *Cpt-Ib* (isoform of *Cpt-I* expressed in muscle) was one out of three genes that showed enhanced expression at the mRNA level after TTA administration (p = 0.003; Table 4). It was also induced by FO (p = 0.003). Also *Cact* mRNA, encoding the protein carnitine-acylcarnitine translocase, was upregulated after TTA treatment (p = 0.002). The *Ucp3* gene (encoding the uncoupling protein 3) was upregulated (p < 0.001), while *Ucp2* (p = 0.008), *Pparδ* (p = 0.002), and *Pparγ* (p < 0.001) were decreased after TTA administration. *Fatp1* (encoding FA transport protein 1, involved in myocardial FA uptake) showed increased expression after FO supplementation (p = 0.001). There was no change in mRNA levels of *PPARα* following treatment with any supplement.

**Table 4:** **Gene expression of selected genes in heart of rats after 50 weeks of diet administration**^**a**^

		**Dietary supplementation**^**b**^			**Statistical significance of variance ratio (P)**^**c**^**, effects of**		
	**Control**	**TTA**	**FO**	**TTA + FO**	**TTA**	**FO**	TTA*FO
Cpt-Ib	1.00 ± 0.17^d^	1.29 ± 0.17	1.29 ± 0.19	1.34 ± 0.14	0.003	0.003	0.04
Ucp2	1.00 ± 0.17	0.92 ± 0.29	1.04 ± 0.17	0.74 ± 0.18	0.008	0.29	0.11
Ucp3	1.00 ± 0.43	2.08 ± 0.76	1.74 ± 0.50	2.31 ± 0.62	<0.001	0.01	0.18
Pgc1α	1.00 ± 0.10	0.99 ± 0.16	1.04 ± 0.19	1.05 ± 0.21	0.95	0.36	0.85
Tfam	1.00 ± 0.08	0.99 ± 0.10	0.98 ± 0.11	0.90 ± 0.15	0.23	0.11	0.33
Nrf1	1.00 ± 0.14	1.06 ± 0.11	1.21 ± 0.12	0.98 ± 0.16	0.06	0.13	0.002
Cd36	1.00 ± 0.14	0.94 ± 0.13	1.09 ± 0.24	0.99 ± 0.10	0.14	0.20	0.68
Cact	1.00 ± 0.14	1.26 ± 0.30	1.18 ± 0.17	1.37 ± 0.22	0.002	0.04	0.61
Fabp3	1.00 ± 0.10	1.15 ± 0.37	1.23 ± 0.17	1.24 ± 0.14	0.26	0.03	0.32
Acads	1.00 ± 1.14	2.31 ± 1.69	1.92 ± 1.91	0.83 ± 1.22	0.82	0.56	0.02
Acadm	1.00 ± 0.18	1.15 ± 0.28	1.14 ± 0.28	1.13 ± 0.26	0.43	0.45	0.32
Acadvl	1.00 ± 0.15	1.12 ± 0.20	1.14 ± 0.16	1.05 ± 0.34	0.80	0.64	0.15
Pparα	1.00 ± 0.34	0.88 ± 0.14	1.15 ± 0.33	1.03 ± 0.20	0.16	0.09	0.97
Pparδ	1.00 ± 0.18	0.87 ± 0.09	1.12 ± 0.17	0.90 ± 0.19	0.002	0.15	0.39
Pparγ	1.00 ± 0.28	0.83 ± 0.16	1.33 ± 0.34	0.87 ± 0.17	<0.001	0.02	0.07
Fatp1	1.00 ± 0.28	1.25 ± 0.25	1.55 ± 0.53	1.50 ± 0.31	0.39	0.001	0.19

Abbreviations: TTA, tetradecylthioacetic acid; FO, fish oil; *Cpt-Ib*, carnitine palmitoyltransferase Ib; *Ucp2*, uncoupling protein 2; *Ucp3*, uncoupling protein 3; *Pgc1α*, peroxisome proliferative activated receptor (gamma coactivator 1); *Tfam*, transcription factor A mitochondrial; *Nrf1*, nuclear respiratory factor; *Cd36*, cluster of differentiation 36; *Cact*, carnitine-acylcarnitine translocase; *Fabp3*, fatty acid binding protein 3; *Acads*, acyl-CoA dehydrogenase short chain; *Acadm*, acyl-CoA dehydrogenase medium chain; *Acadvl*, acyl-CoA dehydrogenase very long chain; *Pparα*, peroxisome proliferator-activated receptor α; *Pparδ*, peroxisome proliferator-activated receptor d; *Pparγ*, peroxisome proliferator-activated receptor γ; *Fatp1*, fatty acid transport protein 1.

^a^ Relative to control diet.

^b^ n=10 in each group.

^c^ P-values from two-way ANOVA.

^d^ Values are mean ± SD.

## Discussion

Long-term treatment with the pan-PPAR agonist TTA or high-dose FO induced marked changes in PUFA composition in heart muscle, resulting in decreased n-6 PUFA and increased n-3 PUFA. The significantly increased levels of EPA, DPAn-3, and DHA in the heart following TTA treatment was the opposite of what has previously been seen in plasma [29] and as shown in liver. The activity of enzymes involved in FA metabolism were also changed in the heart after TTA treatment, including increased CPT-II, ACOX, GPAT, and FAS activities as well as a significant upregulation of *Ucp3* and *Cact* at the mRNA level.

There was no change in expression of *Cd36Fabp3*, or *Fatp1* mRNA, nor a change in TAG concentration, which gives no indication of enhanced entry of FA into the heart. Moreover, as TTA is reported to decrease the n-3 PUFA content in very low density lipoprotein (VLDL) particles [30], a selective increased secretion of n-3 PUFA from liver to plasma is unlikely. Dietary n-3 PUFA has been shown to decrease ARA in rat cardiac mitochondrial phospholipids [31], which is in accordance with the present study. While dietary FO decreased almost all n-6 PUFA in heart, TTA led to a specific decrease in ARA and at the same time an increase in most other n-6 PUFA. Both TTA and FO induced an increase in cardiac EPA and DHA. However, the increase in total n-3 PUFA as a TTA effect was particularly evident for DPAn-3, while cardiac DPAn-3 was decreased after FO treatment. Based on a previous study in platelets, ARA was shunted to the lipoxygenase pathway in the presence of DPAn-3 [32]. Thus, an apparent link exists between ARA and DPAn-3, which might explain part of the mechanisms which underlie decreased ARA and increased DPAn-3 as exerted by TTA. These TTA effects were specific for heart muscle. Similarly, the PPARα-agonist clofibrate has previously been demonstrated to have profound effects on myocardial FA composition, including reduced ARA and increased DHA [26].

TTA induces proliferation of mitochondria and peroxisomes in the liver [33]. Although there were no changes in the gene expression of *Pgc1αTfam* or *Nrf1* in heart after TTA administration, a significant increase in activity of CPT-II and gene expression of *Cpt-Ib* and *Cact* was observed. Furthermore, the peroxisomal β-oxidation system seemed to be induced as the ACOX activity was significantly increased both after TTA and FO administration. These findings suggest that TTA induces both mitochondrial and peroxisomal β-oxidation in heart, supported by two previous studies demonstrating increased myocardial FA oxidation by TTA treatment, associated with reduced cardiac efficiency [22,27]. It has also been shown that TTA induces both CPT-II and ACOX in liver, and to an even larger extent compared to what we report in heart [28]. TTA treatment also resulted in increased activities of cardiac GPAT and FAS. There was also increased enzyme activity of GPAT after FO treatment. This enzyme catalyzes the synthesis of lysophosphatidic acid from glycerol-3-phosphate and long-chain acyl-CoA [34]. Thus, the portion of activated acyl-CoA that is not used for mitochondrial β-oxidation will be esterified into TAG and other glycerolipids, including phospholipids. The increased lipogenesis and TAG biosynthesis together with stimulated FA catabolism can thus explain the enrichment of n-3 PUFA in the heart both after TTA and FO treatment. n-3 PUFA, which are poorly oxidizable FA substrates compared to SFA [35], will most likely be diverted towards phospholipid synthesis and not β-oxidation. This could render n-3 PUFA less available for further metabolism. Altogether, an increased incorporation of n-3 PUFA into phospholipids in heart and a probably increased metabolism of n-3 PUFA in liver due to an extensive increase in CPT-II and ACOX [28] could explain why n-3 PUFA levels increase in heart and decrease in liver and plasma following TTA treatment. Together with the previously mentioned unfavorable metabolic effects of excess PPAR activation [5,22], we consider the possibility that the observed increase of cardiac n-3 PUFA in the current study might not be solely beneficial.

We demonstrated a decrease in CPT-I, but at the same time an increased CPT-II activity in heart, which is in accordance with earlier results on TTA administration in liver [36]. Although CPT-I has been assumed to be rate-limiting for FA oxidation in all tissues, a study has shown that about 50% of CPT-I activity can be inhibited before affecting the actual flux of β-oxidation in the heart [37]. In addition, the present study showed that TTA affected CPT-I activity in the presence of malonyl-CoA by decreasing the sensitivity against this natural inhibitor of CPT-I [38]. However, the reduced CPT-I activity could contribute to an inadequate transport or oxidation of long-chain FA in heart, while shorter FA that do not depend on CPT-I are completely oxidized in the mitochondria.

Expression of uncoupling protein *Ucp3* mRNA is upregulated by PPAR activation, which is consistent with our current findings in the heart, with a strong significant increase after TTA and borderline significant increase after FO administration. This may lead to an increased FA transport and β-oxidation. Although its exact function remains largely unknown, UCP3 could act as an antioxidant and increased *Ucp3* mRNA expression might be a response to an increase in oxidative stress [39]. A previous study associated increased cardiac levels of UCP3 to decreased efficiency in rat heart after high fat feeding, most likely due to enhanced mitochondrial uncoupling [40].

The marked changes on FA metabolism induced by TTA could possibly result in essential FA deficiency due to the decreased levels of n-3 PUFA in liver and plasma [28]. In support of this, TTA treatment was associated with elevated levels of MA both in heart and liver [41]. A metabolic switch inducing the conversion of MA occurs as a result of low dietary levels of linoleic acid (LA) and α-linolenic acid (ALA). Such signs of essential FA deficiency have been reported in animals on a partially hydrogenated FO diet [42] or after treatment with fenofibrate [43], and may be caused by excess PPAR activation. Even though there was no change in *PPARα* mRNA after treatment with TTA or FO, we assume that the effects were primarily due to activation of PPARα. Increased expression of PPARα target genes following TTA [21] and FO [44] treatment have previously been shown in rodent models.

A beneficial effect of n-3 PUFA supplementation has recently been demonstrated in patients with reduced ventricular function and heart failure [14]. Could the observed TTA effects on n-3 PUFA distribution in liver and heart tell us why n-3 PUFA supplements give favorable effects under certain conditions? There might be a link from the n-3 PUFA levels in heart to the association between TTA treatment and reduced cardiac efficiency in normal but not in diabetic mice [22,27]. Furthermore, FO shows some similar effects to TTA on cardiac metabolism, perhaps through PPARα activating mechanisms, which might indicate detrimental effects by giving high doses. However, PPAR independent effects of TTA cannot be excluded. Whether the observed increase of n-3 PUFA in the hearts of rats treated with TTA and FO might also be related to PPAR effects in humans remain to be shown. Additionally, the effect of TTA on PUFA composition in the heart was directly associated with its TTA concentration (data not shown). It is also notable that PPARα and its agonists hold important properties beyond FA and glucose metabolism, like effects on amino acid metabolism [45]. These aspects open for more extensive investigations of PPAR stimulation and cardiac metabolism.

## Conclusions

In summary, long-term administration of the pan-PPAR agonist TTA or high-dose FO to rats, were associated with distinct effects on lipid metabolism in the heart and systemically. FA composition was changed, including a selective increase in n-3 PUFA in rat heart muscle, indicating a limited capacity of heart to metabolize the poorly oxidizable n-3 PUFA compared to SFA. Further, cardiac mitochondrial and peroxisomal FA oxidation seemed to be increased, together with a possible increased mitochondrial uncoupling after TTA treatment. FO in high doses could give similar effects to TTA in heart. These findings might altogether indicate a reduction in cardiac efficiency and are relevant for further studies on the effects of excess PPAR activation in relation to myocardial dysfunction development.

## Methods

### Study design

The animal experiments were standardized according to the Guidelines for the Care and Use of Experimental Animals, and the protocol was approved by the Norwegian State Board for Biological Experiments with Living Animals.

Male Wistar rats, aged eight to ten weeks, were obtained from Taconic Europe A/S (previously Möllegaard and Blomholtgaard, Ry, Denmark). They were housed in groups of five and maintained at a constant 12 h light–dark cycle at a temperature of 22 ± 1°C and a relative humidity of 55 ± 5%. Animals were acclimatized under these conditions for one week prior to study start and had free access to standard chow during the acclimation period and water at all times. During the feeding period the animals received one out of the following four diets for a period of 50 weeks: control diet with 25% (w/v) fat (23% w/v lard, 2% w/v soybean oil); diet supplemented with TTA (0.375% w/v TTA, 22.6 w/v lard, 2% w/v soybean oil); diet supplemented with FO (10.4% w/v EPAX 4020 TG, 12.6% w/v lard, 2% w/v soybean oil); or diet supplemented with TTA and FO (0.375% w/v TTA, 10.4% w/v EPAX 4020 TG, 12.2 w/v lard, 2% w/v soybean oil). These were complete diets containing 20% (w/v) protein from casein from bovine milk (Tine, Tolga, Norway). Lard (Ten Kate Vetten BV, Musselkanaal, Netherlands) and soybean oil (Dyets Inc., Bethlehem, PA, USA) were used as fat sources. Other ingredients were cornstarch, sucrose, fiber, AIN-93 G mineral mix, AIN-93 vitamin mix, L-cysteine, choline bitartrate (Dyets Inc.), and tert-butyl-hydroquinone (Sigma-Aldrich). FO was provided by EPAX AS. TTA was synthesized as previously described [46].

These animals were part of a larger study [47], where they all underwent a jejuno-gastric reflux surgical procedure. A separate experiment enduring 11 weeks were done comparing animals with and without operation, to make sure that the procedure did not affect the nutritional state in the animals. There was no difference in body weight or plasma lipids between the two groups (data not shown), and thus it can be assumed that the operation had no adverse effects regarding nutrition.

### Sampling protocol

The animals were sacrificed with isoflurane (Forene, Abbott Laboratories, Abbott Park, IL) under non-fasting conditions. The abdomen was opened in the midline and blood was drawn by cardiac puncture and collected in BD Vacutainer tubes containing EDTA (Becton, Dickinson, and Company, Plymouth, UK). The heart and liver tissues were collected and immediately freeze-clamped as drainage of blood from the animal was complete. Plasma and tissue samples were stored at −80°C until analyses.

### Quantification of lipids and fatty acids

Tissue samples were homogenized and lipids extracted with chloroform-methanol [48]. Samples were evaporated under nitrogen and redissolved in isopropanol before analysis. Lipids were measured on the Hitachi 917 system (Roche Diagnostics, GmbH, Mannheim, Germany). Total cholesterol (CHOD-PAP) and TAG (GPO-PAP) kits were from Roche Diagnostics and the phospholipids kit from DiaSys Diagnostic Systems GmbH (Holzheim, Germany). FA methyl esters (FAME) were obtained by heating of lipids with methanol at 90°C for one hour. Sulphuric acid was used as a catalyst [49]. After extraction into an organic solvent, FAME were analyzed by gas–liquid chromatography (GC). The gas chromatograph (GC 8000 TOP, Finnigan, USA) was equipped with a programmed temperature vaporization injector, flame-ionization detector, AS 800 autosampler, and with a fused silica capillary column DB1-ms (J & W Scientific, USA). Hydrogen was used as a carrier gas. Column temperature was programmed from 110 to 310°C with a gradient of 2.5°C/min. GC signal was acquired and evaluated with Chromeleon software (Dionex Corporation, USA). Peaks were identified by means of known FA standards and by means of mass spectra, obtained by GC/MS analysis (GCQ, Finnigan, USA) on the same column. Internal standard (C21:0) was used for quantification after calibration with known mixtures of FA standards. FA composition was presented as percentage by weight (wt%). The anti-inflammatory index was calculated using the formula: ((C22:6n-3 + C22:5n-3 + C20:3n-6 + C20:5n-3)/C20:4n-6)*100 [50].

### Enzyme activities

Heart tissue samples were homogenized and fractionated as previously described [23]. The activities of CPT-I and -II [24], ACOX [51,52], GPAT [53], and FAS [54] were measured in the post-nuclear extracts.

### Gene expression analyses

Total cellular RNA was purified from heart tissue using the RNeasy kit and the protocol for fibrous tissue (Qiagen GmbH, Hilden, Germany). RNA quantity was determined spectrophotometrically (NanoDrop 1000, NanoDrop products, Wilmington, DE, USA), while quality was evaluated by capillary electrophoresis (Agilent 2100 Bioanalyzer, Agilent Technologies Inc., Santa Clara, CA, USA) prior to gene analyses. RNA was reversely transcribed to cDNA in 100 μl reactions using TaqMan® Reverse Transcription Reagents (Applied Biosystems, Foster City, CA, USA). Samples were treated with RNase inhibitors as part of the protocol. Selected genes were analyzed using qPCR. The qPCR was performed with ABI PRISM 7900 HT Sequence Detection System (Applied Biosystems). The following 16 genes of interest were selected: Rn00566242 (*Cpt-Ib*), Rn00571166 (*Ucp2*), Rn00565874 (*Ucp3*), Rn00580241 (*Pgc1α*), Rn00580051_m1 (*Tfam*), Rn01455958_m1 (*Nrf1*), Rn00580728 (*Cd36*), Rn00588652 (*Cact*), Rn00577366 (*Fabp3*), Rn00563649 (*Acadvl*), Rn00566390 (*Acadm*), Rn00574634 (*Acads*), Rn00566193 (*Pparα*), Rn00565707 (*Pparδ*), Rn00440945 (*Pparγ*), and Rn00585821_m1 (*Fatp1*). All primer/probe sequences for the genes studied were obtained from Applied Biosystems. The MIQE guidelines for qPCR analyses were used when selecting house-keeping genes [55,56]. Out of the three house-keeping genes originally included in the analyses: Hs99999901_s1 (*18 S*, Eurogentec S.A., Seraing, Belgium), Rn99999916_s1 (*Gapdh*, Applied Biosystems), and Rn00821065_g1 (*Arbp*, Applied Biosystems), *18 S* was found to be the best using geNorm [57]. This endogenous house-keeping gene was used to normalize the expression value of each gene in all samples.

### Statistical analyses

The results were presented as means with their standard deviations (SD) for a minimum of eight and a maximum of fifteen rats per group. Gene expression data was normalized against the control diet group. Data was evaluated by two-way ANOVA for treatment additivity and synergy [58]. Results were not adjusted for multiple comparisons, and thus p-values <0.01 were considered significant. Statistics were performed by using PASW Statistics for Windows, version 18 (SPSS Inc., Chicago, IL, USA).

## Abbreviations

ACOX, Fatty acyl-CoA oxidase; ALA, α-linolenic acid; ANOVA, Analysis of variance; ARA, Arachidonic acid; CACT, Carnitine-acylcarnitine translocase; CD36, Cluster of differentiation 36; CoA, Coenzyme A; CPT, Carnitine palmitoyltransferase; DHA, Docosahexaenoic acid; DPAn-3, Docosapentaenoic acid (n-3); EPA, Eicosapentaenoic acid; FA, Fatty acid; FABP, Fatty acid binding protein; FAME, Fatty acid methyl ester; FAS, Fatty acid synthase; FATP, Fatty acid transport protein; FO, Fish oil; GC, Gas–liquid chromatography; GPAT, Glycerol-3-phosphate acyltransferase; LA, Linoleic acid; MA, Mead acid; MUFA, Monounsaturated fatty acids; PPAR, Peroxisome proliferator-activated receptor; PUFA, Polyunsaturated fatty acids; qPCR, Real-time PCR; SFA, Saturated fatty acids; TAG, Triacylglycerol; TTA, Tetradecylthioacetic acid; UCP, Uncoupling protein; VLDL, Very low density lipoprotein1.

## Competing interests

The authors declare that they have no competing interests.

## Authors’ contributions

The authors’ responsibilities were as follows: ON, KB, HW, AV, and RKB: developed the study concept and design and supervised the study; ON, KB, HW, AV, and RKB: obtained funding; ES, CB, PB, BJC, KB, and HW: collected materials and data; ES, BB, ON, LB, PB, KB, HW, AV, and RKB: provided administrative, technical and material support; ES, BB, ON, LB, PB, and RKB: analyzed and interpreted data; ES: conducted the statistical analysis; ES: drafted the manuscript, including figures and tables; and ES, BB, ON, LB, CB, PB, BJC, KB, HW, AV, and RKB: contributed to the critical revision of the manuscript. All authors read and approved the final manuscript.

## Supplementary Material

Additional file 1 PUFA composition (wt%) in heart of rats after 50 weeks of diet administration. Click here for file
