# Supplementary material for: Long-term treatment with the pan-PPAR agonist tetradecylthioacetic acid or fish oil is associated with increased cardiac content of n-3 fatty acids in rat
Source: Lipids Health Dis. 2012 Jun 27;11:82. doi: 10.1186/1476-511X-11-82 (PMC3459737; doi:10.1186/1476-511X-11-82)
Supplement: Additional file 1 — PUFA composition (wt%) in heart of rats after 50 weeks of diet administration. [file 1476-511X-11-82-S1.doc]

| **Supplement A PUFA composition (wt%) in heart of rats after 50 weeks of diet administration** | | | | | | | | |
| --- | --- | --- | --- | --- | --- | --- | --- | --- |
|  | **Dietary supplementation^a^** | | | |  | **Statistical significance of**  **variance ratio (P)^b^, effects of** | | |
|  | **Control** | **TTA** | **FO** | **TTA + FO** |  | **TTA** | **FO** | **TTA*FO** |
| **n-6 PUFA** | 41.8 ± 2.4^c^ | 34.9 ± 2.1 | 23.8 ± 2.2 | 12.8 ± 2.4 |  | <0.001 | <0.001 | 0.003 |
| **C18:2n-6 (LA)** | 15.7 ± 1.0 | 17.2 ± 2.0 | 9.2 ± 1.0 | 5.8 ± 1.2 |  | 0.03 | <0.001 | <0.001 |
| **C18:3n-6** | 0.02 ± 0.01 | 0.05 ± 0.01 | 0.02 ± 0.01 | 0.04 ± 0.01 |  | <0.001 | 0.26 | 0.13 |
| **C20:2n-6** | 0.24 ± 0.01 | 0.33 ± 0.05 | 0.22 ± 0.02 | 0.23 ± 0.02 |  | <0.001 | <0.001 | <0.001 |
| **C20:3n-6** | 0.49 ± 0.06 | 1.36 ± 0.14 | 0.36 ± 0.04 | 0.55 ± 0.07 |  | <0.001 | <0.001 | <0.001 |
| **C20:4n-6 (ARA)** | 23.8 ± 2.5 | 13.2 ± 1.6 | 13.7 ± 2.2 | 5.9 ± 1.4 |  | <0.001 | <0.001 | 0.02 |
| **C22:2n-6** | 5.0 ± 5.2^d^ | 3.3 ± 4.9^d^ | 1.7 ± 3.9^d^ | 0.8 ± 2.9^d^ |  | 0.32 | 0.02 | 0.74 |
| **C22:4n-6** | 0.82 ± 0.09 | 1.32 ± 0.32 | 0.08 ± 0.01 | 0.06 ± 0.01 |  | <0.001 | <0.001 | <0.001 |
| **C22:5n-6** | 0.81 ± 0.16 | 1.43 ± 0.34 | 0.25 ± 0.03 | 0.20 ± 0.05 |  | <0.001 | <0.001 | <0.001 |
| **n-3 PUFA** | 16.0 ± 2.0 | 19.0 ± 3.8 | 36.7 ± 3.3 | 47.8 ± 4.0 |  | <0.001 | <0.001 | <0.001 |
| **C18:3n-3 (ALA)** | 0.15 ± 0.06 | 0.20 ± 0.06 | 0.14 ± 0.06 | 0.10 ± 0.06 |  | 0.57 | 0.004 | 0.02 |
| **C18:4n-3** | 0.4 ± 0.3^d^ | 0.3 ± 0.3^d^ | 82.8 ± 38.0^d^ | 89.4 ± 30.4^d^ |  | 0.64 | <0.001 | 0.64 |
| **C20:4n-3** | 0.05 ± 0.01 | 0.04 ± 0.00 | 0.14 ± 0.02 | 0.13 ± 0.02 |  | 0.02 | <0.001 | 0.18 |
| **C20:5n-3 (EPA)** | 0.12 ± 0.01 | 0.17 ± 0.03 | 3.5 ± 0.6 | 4.5 ± 0.6 |  | <0.001 | <0.001 | <0.001 |
| **C21:5n-3** | 0.2 ± 0.2^d^ | 0.2 ± 0.1^d^ | 27.0 ± 11.8^d^ | 21.6 ± 8.4^d^ |  | 0.20 | <0.001 | 0.21 |
| **C22:5n-3 (DPAn-3)** | 3.0 ± 0.4 | 4.9 ± 1.1 | 1.8 ± 0.1 | 2.0 ± 0.2 |  | <0.001 | <0.001 | <0.001 |
| **C22:6n-3 (DHA)** | 12.8 ± 1.7 | 13.6 ± 3.7 | 31.1 ± 3.2 | 41.0 ± 3.8 |  | <0.001 | <0.001 | <0.001 |

Abbreviations: TTA, tetradecylthioacetic acid; FO, fish oil; FA, fatty acids; PUFA, polyunsaturated fatty acids; LA, linoleic acid; ARA, arachidonic acid; ALA, α-linolenic acid; EPA, eicosapentaenoic acid; DPAn-3, docosapentaenoic acid (n-3); DHA, docosahexaenoic acid

^a^ n=12 in each group

^b^ P-values from two-way ANOVA

^c^ Values are mean ± SD

^d^ Values are *10^-3^
